# Assessing the Influence of Betaine-Based Natural Deep Eutectic Systems on Horseradish Peroxidase

**DOI:** 10.1021/acssuschemeng.2c04045

**Published:** 2022-09-12

**Authors:** Nicolás F. Gajardo-Parra, Liane Meneses, Ana Rita C. Duarte, Alexandre Paiva, Christoph Held

**Affiliations:** † Laboratory of Thermodynamics, Department of Biochemical and Chemical Engineering, TU Dortmund University, Emil-Figge-Str. 70, 44227 Dortmund, Germany; ‡ LAQV-REQUIMTE, Department of Chemistry, School of Science and Technology, NOVA University Lisbon, 2825-149 Caparica, Portugal

**Keywords:** biocatalysis, thermodynamics, natural deep eutectic systems (NADES), perturbed-chain statistical associating fluid theory (PC-SAFT), viscosity, density, water activity

## Abstract

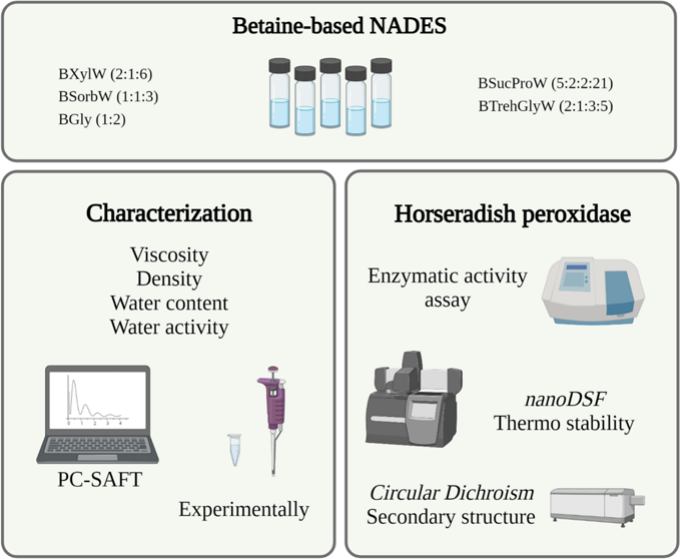

To validate the use of horseradish peroxidase (HRP) in natural deep eutectic systems (NADES), five different betaine-based NADES were characterized in terms of water content, water activity, density, and viscosity experimentally and by thermodynamic modeling. The results show that the NADES under study have a water activity of about 0.4 at 37 °C for water contents between 14 and 22 wt %. The densities of the studied NADES had values between 1.2 and 1.3 g^.^cm^–3^ at 20 °C. The density was modeled with a state-of-the-art equation of state; an excellent agreement with the experimental density data was achieved, allowing reasonable predictions for water activities. The system betaine:glycerol (1:2) was found to be the most viscous with a dynamic viscosity of ∼600 mPa^.^s at 40 °C, while all the other systems had viscosities <350 mPa^.^s at 40 °C. The impact of the NADES on the enzymatic activity, as well as on, conformational and thermal stability was assessed. The system betaine/sorbitol:water (1:1:3) showed the highest benefit for enzymatic activity, increasing it by two-folds. Moreover, upon NADES addition, thermal stability was increased followed by an increment in a-helix secondary structure content.

## Abstract 2

In this work, the influence of five betaine-based natural deep eutectic systems on the activity and conformation of horseradish peroxidase was studied. The important conclusion is that binary parameters that were fitted to density were able to predict *a*_*w*_ values successfully.

## 1 Introduction

Horseradish peroxidase (HRP) is an oxidoreductase enzyme (E.C. 1.11.1.7) present in the roots of the perennial herb, produced on a large scale due to its industrial application in clinical diagnostic kits or immunoassays.^1,2^ Although HRP is a considerably thermostable enzyme, its structural stability and biocatalytic activity are essential for the inclusion in industrial processes.^3^ Various approaches have been used in the literature to increase enzyme activity without decreasing stability, such as protein engineering, high-pressure operation, protein immobilization, and the addition of co-solvents.^4^

Among the green solvents studied, deep eutectic systems (DES) are prominent homogeneou liquids solvents obtained from the mixture of two or more components that, in a particular molar ratio show a pronounced decrease in the melting point due to strong interactions.^5^ When all the components used are naturally occurring products they are categorized as natural deep eutectic systems (NADES).^6^ DES and NADES have been applied in numerous engineering fields.^6^ Specifically, NADES have been used in several enzymatic reactions, either as a co-solvent or as reaction medium.^7^ NADES had positive effects on the reaction kinetics of bovine live catalase,^8^ boost the enzymatic activity of laccases,^9^ improved the stability of lipases,^10^ and increased the yield of oxidoreductases,^11^ among others. Nevertheless, in most cases, the election of an appropriate NADES for the reaction lacks theoretical explanation and is based on trial-and-error procedures.

Water activity (*a*_w_) is one of the parameters that highly influences enzymatic activity, and several authors have studied its influence. Water is necessary to ensure enzymatic mobility; nevertheless, in excess it can also promote interactions that could change the enzyme confirmation, which can be harmful due to the complete loss of the structure.^12^ Enzyme conformation during storage and reaction depends on an essential hydration shell, which acts as a lubricant that allows conformational mobility and molecular environment adaptation.^13^ A reaction environment with controlled *a*_w_ can also positively affect the enzyme thermostability, preventing heat inactivation.^14^ Knowledge of *a*_w_ of the NADES allows us to design media for enzyme storage and stabilization without compromising their enzymatic activity due to the inadequate moisture content.

Bioprocesses significantly benefit from predictive methods that substantially reduce the number of required trial and error experiments.^15^ The above-described important property, *a*_w_, can be accessed from predictive methods. Equations of state are particularly promising due to the ability to describe densities and activity coefficients by taking into account explicit molecular interactions.^16^ Perturbed-Chain Statistical Associating Fluid Theory (PC-SAFT) has proven to be capable of predicting physicochemical properties of different compounds and mixtures.^17,18^ PC-SAFT calculations have been successful for systems of varying complexity such as amino acids,^19^ aromatics,^20^ electrolytes,^21^ proteins,^22^ ionic liquids, and deep eutectic solvents.^23,24^ Specifically, Zubeir et al. proposed the individual-component approach for modeling of DES.^25^ This approach provides the flexibility to screen physical properties from DES based on their hydrogen bond acceptors (HBAs) and hydrogen bond donors (HBDs) constituents as a function of molar ratios using only pure-component parameters of HBAs and HBD.

Enhanced enzyme activity and stability toward solvent and temperature are desired in industrial processes to enable a broader process-operation window for more flexible processes and potentially improved efficiencies.^26^ Liquid-phase conditions such as pressure, pH, *a*_w_, ionic strength, and the addition of co-solvents are known to affect the stability of an enzyme.^27^ Co-solvents influence enzymatic stability by modifying the chemical structure, polarity, viscosity, and the ability to build hydrogen bonds with the protein surface.^28^ Thermal stability is often represented by the temperature at which 50% of a protein is folded. In contrast, structural stability is often characterized by changes in the conformation of protein secondary major structures.^29,30^ Various methods such as differential scanning calorimetry, circular dichroism (CD), or differential scanning fluorometry (DSF) can be used to quantify the temperature at which the protein changes from an entirely folded native state to an unfolded state.^31−33^ Previous studies on HRP have studied the effects of pH,^34,35^ and the addition of co-solvents such as ionic liquids,^36^ sugars,^37^ and ammonium salts^38^ on thermal stability and also on enzymatic activity.^39,40^ Co-solvents stabilize proteins mainly by the excluded volume effect, while some sugars such as trehalose, sucrose, and sorbitol use enthalpic interactions with amino acids from the protein backbone that can lead to a decrease in enzymatic activity for high sugar concentrations. Additive effects in co-solvent mixtures have been previously reported by Jaworek et al., suggesting that protein stability can benefit from both the exclusion effect and enthalpic interactions.^41^

In this work, we proposed the use of betaine-based NADES to improve the enzymatic activity and stability of HRP. Five different NADES were characterized by measuring density, viscosity, and *a*_*w*_. These properties were obtained experimentally, as well as modeled, using PC-SAFT. nanoDSF was used to obtain the denaturation temperature to evaluate the effect of NADES on the thermal stability of HRP, and CD was used to quantify the conformational changes in the secondary structure of HRP. Boosting enzyme stability and activity using green solvents enhances the possibility of using these technologies in industrial processes.

## 2 Materials and Methods

### 2.1 Chemicals

Lyophilized powder of peroxidase from HRP, (type I, 89.63 U/mg solid, CAS 9003-99-0) was purchased from Sigma-Aldrich (St. Louis, Missouri, USA) and used without further purification. D-(+)-xylose (≥99%, CAS 58-86-6), glycerol (≥99.5% CAS 56-81-5), D-sorbitol (≥98%, CAS 50-70-4), DL-proline (99%, CAS 609-36-9), phenol-4-sulfonic acid sodium salt dihydrate (PSA, 98%, CAS 10580-19-5), 4-aminoantipyrine (4-AAP, ≥ 99%, CAS 83-07-8) and hydrogen peroxide 30% solution (CAS 7722-84-1) were purchased from Sigma-Aldrich (St. Louis, Missouri, USA). Trehalose dihydrate (CAS 6138-23-4) was kindly provided by Hayashibara Co., LDA (Okayama, Japan). Betaine anhydrous (>97%, CAS 107-43-7) was obtained from TCI (Tokyo, Japan) and sucrose (CAS 57-50-1) was purchased from Cmd Chemicals (Funchal, Portugal).

### 2.2 NADES Preparation and Water Concentrations

Five systems were prepared using betaine as HBA, with the HBDs: xylose, trehalose, sucrose, proline, or glycerol. All NADES used in this work were prepared gravimetrically using the heating-and-stirring method described elsewhere.^42^ The systems prepared are listed in Table 1. To further study the influence of different water contents on the properties of the NADES and HRP conformation, BGly + water mixtures were prepared with varying contents of water, to obtain 95, 90, 85 and 80 wt % BGly.

**Table 1:** Components Used to Prepare the Systems Used in This Work With the Respective Molar Ratios and Code Names^a^

code	molar ratio	component A	component B	component C	component D	reference
BXylW	2:1:6	betaine anhydrous	D-(+)-xylose	water		Jesus, 2021
BTrehGlyW	2:1:3:5	betaine anhydrous	trehalose dihydrate	glycerol	water	Jesus, 2021
BSorbW	1:1:3	betaine anhydrous	D-sorbitol	water		this work
BSucProW	5:2:2:21	betaine anhydrous	sucrose	DL-proline	water	Jesus, 2021
BGly*	1:2	betaine anhydrous	glycerol			Rodrigues, 2021

a different weight fractions of water were prepared: 5, 10, 15 and 20 wt % water (95, 90, 85 and 80 wt % BGly, respectively).

### 2.3 NADES Characterization

#### 2.3.1 Water Content and Water Activity

The water content of the systems was determined by Karl-Fisher (KF) titration, performed in an 831 KF Coulometer with the generator electrode without diaphragm, using Hydranal Coulomat AG as a reagent. For each system, the water content was determined in triplicate. The *a*_*w*_ of the systems was determined using a AwTherm–Water Activity meter (Rotronic, Bassersdorf, Switzerland), in equilibrium mode, at 37 and 60 °C. For each system, the *a*_w_ was determined in triplicate.

### 2.4 Density and Viscosity Measurements

The viscosities and the densities of the systems were determined using an Anton Paar SVM 3001 viscometer (Graz, Austria) in a temperature range from 20 to 80 °C (±0.03 °C), with 10 °C steps. The measurements were performed in triplicate for each sample. The pressure of the equipment was 100 kPa, and the uncertainty of density measurements was 0.0002 g^.^cm^–3^.

### 2.5 Enzyme Stability

#### 2.5.1 Enzymatic Activity Assay

HRP in NADES mixtures were prepared by suspending HRP in pure NADES. After that, PBS (100 mM, pH 7) was added, resulting in the dissolution of all the components in PBS, hence obtaining a NADES aqueous solution (NADES-AS), containing 1 mg^.^mL^–1^ HRP, and 20 wt % NADES. The enzymatic activity of HRP in the presence of the NADES was determined using a colorimetric method adapted from Wu et al.^43^ Briefly, in a cuvette, 950 μL of PSA, 950 μL of 4-AAP, and 50 μL of H_2_O_2_, were added to 1 mL of PBS, yielding the final concentrations of 10, 2.4, and 2 mM, respectively. After homogenization, this solution was used as a blank reference. Then, 50 μL of NADES-AS was added, and the increase in the absorbance at 490 nm was followed for 1 min in an UV–vis Genesys 50 spectrophotometer (ThermoFischer Scientific, Waltham USA). The molar extinction coefficient (ε) of 5560 M^.^cm^–3^ was used as determined elsewhere.^1^ The assays were performed in triplicate at 25 °C. The concentration of HRP in NADES-AS was determined using the Lowry method for protein quantification,^44^ using bovine serum albumin (BSA) as standard, in concentrations ranging from 20 to 100 μg^.^mL^–1^ at 25 °C.

### 2.6 Thermal Stability with nanoDSF

In this work, unfolding temperature (*T*^unfolding^) was measured using the nanoDSF apparatus Prometheus NT.48 (NanoTemper, Munich, Germany). The method is based on the difference in measured fluorescence between tryptophan and tyrosine, present in abundance before and after the denaturation process, respectively. For this, the fluorescence ratio F350/F330 is used as previously described in the literature.^41,45,46^ Furthermore, the equipment has a back-reflection technology that detects the aggregation of the sample, by the attenuation of the light that passes through the cell, which is collected from its reflection on the surface of the sample. The equipment is charged with 10 μL of the enzyme + NADES-AS. The HRP concentration was 0.5 μM in PBS (100 mM, pH 7) buffer solution. Measurements were performed for the different NADES-AS of this work at 20 wt % in the buffer solution. For data collection and data processing, software PR.ThermControl, version 2.1.2, was used.

### 2.7 Structural Stability with Circular Dichroism

CD spectra were obtained between 190 and 250 nm in a Chirascan qCD spectrometer (Applied Photophysics, Leatherhead, UK) equipped with a Quantum Northwest TC125 temperature controller. HRPs (5 μM) in NADES-AS (5 wt %) were used to obtain the spectra from 190 to 240 nm, at 25 °C, using a 0.1 mm pathlength. The secondary structure contents were calculated using CONTIN-LL (Provencher & Glockner Method), with reference data set SP175,^47^ in the DICHROWEB web server (http://dichroweb.cryst.bbk.ac.uk).

## 3 Modeling

In 2001, Gross and Sadowski introduced the state-of-the-art thermodynamic equation of state PC-SAFT.^17,18^ In this work, PC-SAFT was used to predict the water influence on thermodynamic properties, specifically the *a*_w_ in NADES. PC-SAFT commonly calculates the residual Helmholtz-energy difference between the total molar energy and the ideal gas energy. The residual energy is calculated as the sum of the contributions of hard-chain repulsion,^48^ dispersion attraction, and site–site bonding interactions, as shown in eq 1.
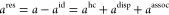
1

A detailed description of each contribution is given elsewhere.^17,18^ Five pure-component parameters are necessary to calculate these contributions for associating molecules: segment number, *m*_i_^seg^, the segment diameter, σ_i_, the dispersion–energy parameter, *u*_i_/*k*_B_, the association–energy parameter, ε^AiBi^/*k*_B_, and the association–volume parameter, κ^AiBi^. Each molecule was characterized separately to describe the contributions in NADES, using the individual-component approach described by Zubeir et al.^25^ For the description of mixtures, the Berthelot-Lorenz combining rules were used for the segment diameter and the dispersion energy, as shown in eqs 2 and 3 where *k*_*ij*_ is an adjustable binary interaction parameter used in this work.
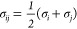
2
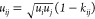
3

The combining rules suggested by Wolbach and Sandler for associative compounds were applied.^49^ Available pure-component parameters and binary interactions parameters were retrieved from the literature. All PC-SAFT parameters used in this work are reported in Table S1. Calculating the *a*_*w*_ requires assessing the water activity coefficients. For this, PC-SAFT was used to determine the water fugacity coefficient in the mixture normalized by the pure-component state, as shown in eq 4
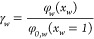
4

## 4 Results and Discussion

### 4.1 Viscosity

**Figure 1:**
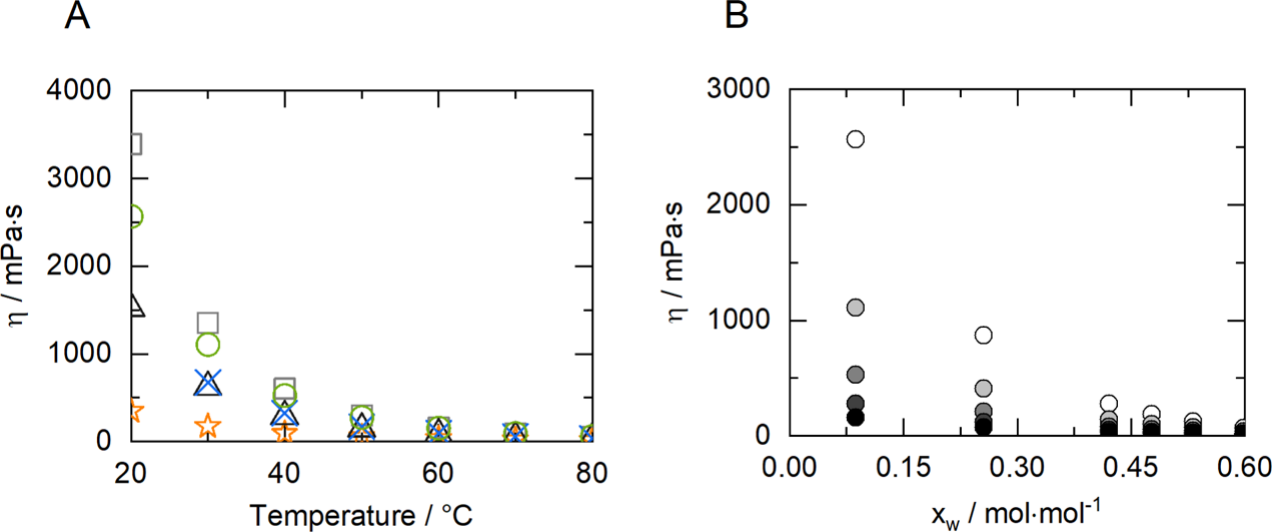
Experimental viscosity (mPa·s) as a function of the temperature for (A) BXylW (stars), BTrehGlyW (squares), BSorbW (triangles), BSucProW (cross), and BGly (circles) and (B) BGly as a function of water mole fraction at different temperatures: 20 °C (white), 30 °C (light gray), 40 °C (gray), 50 °C (dark gray), 60 °C and (black). Data are shown in Table S3 and S4.

The presence of betaine as HBA turns the obtained NADES into highly viscous liquids; it is known from the literature that this is caused by the strong molecular interactions that can affect molecular mobility.^50^ As shown in Figure 1A, BTrehGlyW has the highest viscosity, possibly due to the low flexibility for molecular mobility that hydrogen bond interactions and structures provide among the NADES constituents (betaine, trehalose and glycerol) and water. BSucProW and BSorbW have similar viscosity values. It would be expected that BSucProW, due to the higher complexity of its structure offered by the additional component, would have a higher viscosity than BSorbW. However, due to the higher water content of BSucProW (19.3 wt %) the viscosities of the two NADES almost overlap, as it can be noticed in Figure 1A. Due to the low water content, BGly is one the systems with higher viscosity, while BXylW has the lowest viscosity of the NADES studied. This can be caused by a combination of factors, namely, its simple chemical structure, low density, and higher water content. As expected, the viscosity decreases drastically with the temperature for all NADES, as shown in Table S3.

Side by side with temperature, water addition is known to decrease the viscosity of these systems.^35,36^ We have studied its effect on the system BGly and as shown in Figure 1B, adding 20 wt % water, at 20 °C, reduces the viscosity from ∼2600 mPa·s down to ∼60 mPa·s (the values are listed in Table S4). Although water addition is an essential tool for reducing the viscosity in industrial applications, it is crucial to make sure the non-disruption of the molecular interactions between the HBA and HBD of the NADES, that for choline chloride based NADES starts at around 40% molar of water.^51^ Nevertheless, the exact influence of water concentrations on the behavior of betaine based DESs is not yet known.

### 4.2 Density of NADES

The density of the NADES was determined experimentally in a temperature range from 30 to 80 °C and PC-SAFT was used to model the data. The density of the systems ranged from 1.210 g^.^cm^–3^, for BXylW and BGlyW, to 1.280 g^.^cm^–3^, for BTrehGlyW at 40 °C. These values are similar to other betaine-based systems with polyols reported by Rodrigues et al.^24,52^ Moreover, Kucan et al. studied the density of BGly in a different molar ratio (1:3), which also fell within the range obtained in this study, 1.20 g^.^cm^–3^, at 15 °C and 1.23 g^.^cm^–3^, at 55 °C.^53^ Altamash et al. have also reported the density of NADES combining betaine and other compounds, such as acids, and the values range between 1.2 and 1.3 g^.^cm^–3^.^54^

**Figure 2:**
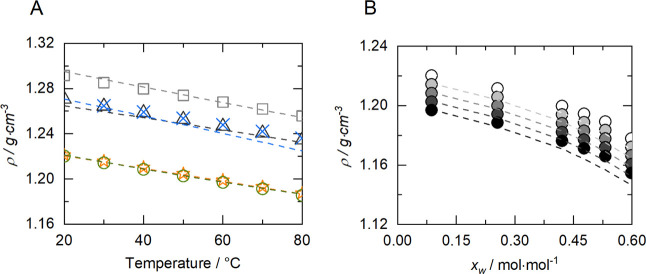
Density (g·cm^–3^) in function of the temperature of (A) BXylW (stars), BTrehGlyW (squares), BSorbW (triangles), BSucProW (cross), and BGly (circles) and (B) BGly as a function of mass fractions of BGly: 100% (white), 95% (light gray), 90% (gray), 85% (dark gray), and 80% (black). Dashed lines represent PC-SAFT calculations with parameters reported in Table S1 and S2. Data are shown in Table S5 and S6.

As expected, the density decreased linearly with increasing temperature for all systems, as shown in Figure 2A. BGly and BXylW have a similar density, which is lower than the other systems under study, although having a significantly different amount of water. On the one hand, the differences in density can be attributed to electrostatic forces and hydrogen bonds between HBA, HBD, and water, which decreases the free volume in the mixture and increases the density. In other words, the more OH groups within the NADES the higher the density.^11,34^ On the other hand, the spatial orientation influences density of NADES due to the steric effect of aromatic groups or large sugars, as in the case of systems comprising xylose. As shown in Figure 2B, water addition causes a decrease in density. Table S5 shows the density values of the all the systems at different temperatures.

The liquid densities for the systems used in this work were modeled with PC-SAFT using the individual-component approach. Although modeling density is straightforward for equations of state, modeling density of NADES is challenging. The reason is that the HBA and HBD are solids^2,12^ except glycerol, and parameters of HBA and HBA could thus not be fitted to density of the pure HBA and HBD in the original references for the parameters of HBA and HBD (see Table S1), respectively. Thus, it was necessary to use binary interaction parameters to correlate the density of the systems under study. The modeling results were within an overall average absolute deviation (AAD) of 0.43%. This is an excellent result, and it shows that fitting binary parameters to experimental density is a valid option. Furthermore, these parameters were used to predict other properties (see the next section).

### 4.3 Water Activity

Since these systems were chosen based on their potential use in biocatalytic applications, determining water activity is quite relevant. The water activity of the systems under study was simultaneously predicted using PC-SAFT and determined experimentally and the results demonstrate that the predicted values are in accordance with the results obtained, validating the model used. First, the experimental data is discussed. The experimental results for water activity at defined water contents of the NADES used in this work are shown in Table 2. Except for BGly, all the NADES needed the addition of water (ranging between 40 and 70 mol %) to be prepared (Table 1). These water mole fractions correspond to water mass fractions between 14 and 22 wt % water, respectively. From the results presented in Table 2, it is possible to observe that the *a*_w_ values of the systems at 37 °C (except for BGly) are *a*_*w*_ ∼ 0.4, despite the mixtures were prepared with very different amounts of water contents. Even though some NADES present high water mass fractions, NMR studies of the NADES herein used and reported elsewhere, prove that in these conditions water is part of the hydrogen bond network that is involved in the formation of the supramolecular structure of the NADES.^55^

**Table 2:** Water Content (wt %) and *a*_w_ at 37 and 60 °C at 100 kPa of the Systems Under Study

system	water content (wt %)	*a*_w_ at 37 °C	*a*_w_ at 60 °C
**BXylW**	21.9 ± 0.2	0.444 ± 0.004	0.469 ± 0.005
**BTrehGlyW**	14.6 ± 0.3	0.394 ± 0.002	0.413 ± 0.005
**BSorbW2**	14.3 ± 0.5	0.433 ± 0.009	0.417 ± 0.008
**BSucProW**	19.3 ± 1.2	0.443 ± 0.001	0.461 ± 0.007
**BGly**	1.7 ± 0.1	0.071 ± 0.004	0.082 ± 0.003

**Figure 3:**
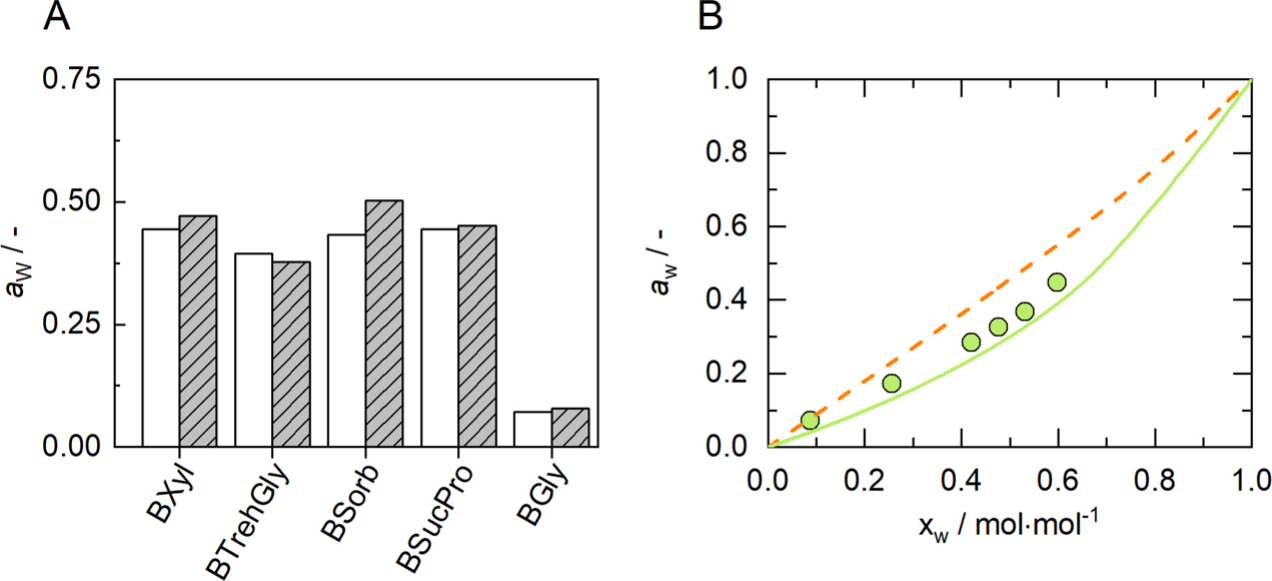
*a*_w_ values at 37 °C and 100 kPa. (A) NADES with compositions in Table 1. Experimental (empty bars), PC-SAFT predictions *k*_*ij*_ = 0 (stripped bars). (B) Water influence on BGly. Experimental data (circles), PC-SAFT predictions with *k*_*ij*_ = 0 between betaine and glycerol (dashed line), PC-SAFT predictions using *k*_ij_ between betaine and glycerol fitted to density (full line). PC-SAFT parameters are reported in Tables S1 and S2.

The NADES BGly contains only residual water (<2 wt %), and, as so, *a*_w_ is lower than that for the other studied NADES. As expected, *a*_w_ increases upon addition of water to BGly, cf. Figure 3B. At the maximum water mole fraction studied (*x*_*w*_ = 0.6, which corresponds to ≈20 wt % water), the *a*_w_ was found to be ≈0.43, which falls within the *a*_w_ values determined for the other systems (cf. Table 2).

It can be further seen from Figure 3B that *a*_w_ and the water mole fraction are different, and the difference is most pronounced at equimolar NADES/water ratio. Furthermore, activity is lower than the mole fraction; that is, activity coefficients of water (γ_*w*_) must be lower than one (*a*_w_ = *x*_w_ γ_w_). The γ_w_ values modeled with PC-SAFT are lower than one for the systems under study; that is, water interactions in the NADES mixtures are more substantial than that in pure water. This is caused by the strong hydrogen bonding of the NADES constituents with water. Figure 3A shows the qualitative agreement for *a*_*w*_ obtained by the predictions with PC-SAFT without using any binary interaction parameters between HBA and HBD. As Baz et al*.*^56^ noticed, the individual-component approach provides flexibility to the model without losing quality in the predictive results, achieving an AAD of 7.76%. However, by increasing the amount of water in the mixture, the HBA-HBD interactions weaken rapidly.^57^ Hence, incorporating a binary interaction parameter increases the accuracy of PC-SAFT modeled *a*_w_ in the BGly + water dilutions, as shown in Figure 3B. It is important to note that for the system BGly one binary parameter between betaine and glycerol was fitted to the independent experimental data (density data, cf. Section 4.2); the availability of this single parameter allows predicting shape of the *a*_*w*_ curve within an AAD of 16.2%, a satisfying agreement from the experimental data with water uptake up to *x*_w_ = 0.6 (cf. Figure 3B).

### 4.4 Influence of NADES on Enzymatic Activity of HRP

**Figure 4:**
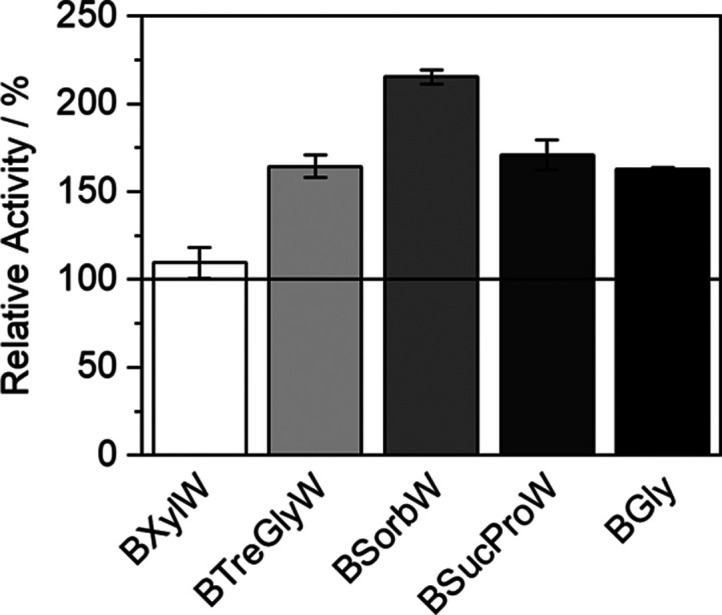
Relative enzymatic activity of HRP in five different NADES-AS using PBS (100 mM, pH 7) as control at 37 °C and 100 kPa.

The enzymatic activity of HRP at 37 °C was studied in NADES-AS, using PBS (100 mM, pH 7) as control. The activity was assessed by a colorimetric method to determine the production of a dye, by the oxidation of PSA in the presence of 4-AAP.^1^Figure 4 illustrates that in all the NADES-AS, there was an increase in the enzymatic activity and reaction rate, compared to the control buffer. The addition of BTrehGlyW, BSucProW, and BGly lead to an increase in the enzymatic activity of approximately 60%. The NADES that caused the highest impact on enzymatic activity was BSorbW, in which the enzymatic activity increased two-fold compared to the control buffer.

In the literature, only the effect of choline-based DES had been studied on HRP; however, discordant results were found. While one study shows the improvement of HRP activity in the presence of DES,^43^ more recent results indicate that HRP’s activity decreased, especially for higher DES concentrations.^58^ Moreover, as recently reviewed, most DES used for protein stabilization and activation are based on choline derivatives.^59^ These two findings were the driven force for the development of this work. On the one hand, it was important to study the impact of NADES on HRP activity and stability. On the other hand, replacing choline chloride in such applications has become urgent due to its hygroscopic behavior, as well as the limitations to its application imposed by several industries. Betaine-based NADES have been used for some preservation ends, such as for protein stabilization^59^ or cryopreservation,^55^ hence this was our starting point for choosing this family of NADES.

In order to understand how NADES influenced HRP’s enzymatic activity, several structural studies were performed, which will now be discussed.

### 4.5 Temperatures of Unfolding

**Figure 5:**
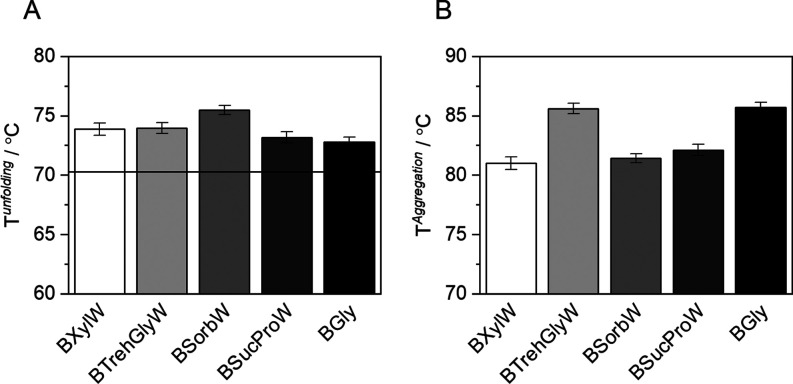
Thermal stability of HRP in the presence of NADES-AS used in this work. (A) Experimental unfolding temperature and (B) aggregation temperature. Experiments carried out at 100 kPa and *p*H 7. The horizontal continuous black line represents the unfolding temperature in neat buffer.

The denaturation temperature was measured to determine the impact of NADES on the protein unfolding process. As thermal stabilization mediated by the co-solvents directly influences unfolding temperature,^60^ it was expected that the NADES used in this work also increase unfolding temperatures (*T*^unfolding^); this could indeed be observed, as shown in Figure 5. Table S7 shows the *T*^unfolding^ and aggregation temperatures (*T*^aggregation^) at ambient pressure, and a heating rate of 0.7 °C·min^–1^. *T*^unfolding^ and *T*^aggregation^ are listed in the control buffer as well as in the different NADES-AS.

HRP follows the denaturation model proposed by Lumir and Eyring,^61^ in which an intermediate state can be observed before unfolding. This intermediate state is determined by the melting of the tertiary structure of the protein near the distal heme group, without significant changes in the secondary structure.^35^ As shown in Table S7 the addition of NADES-AS decreases, in the case of BSorbW and BSucProW considerably, the *T*_on-set_ with respect to the experiment in a neat buffer. This temperature represents the beginning of the protein unfolding, so this result could indicate that NADES-AS promotes the HRP intermediate state coupled with changes in the secondary structure, an effect previously observed in other enzymes.^58,62^ This is accompanied by a slow unfolding, where the enzyme exhibits a boost in activity, ending later than the control in neat buffer. Hydrogen bond-like interactions of the exposed distal heme pocket, at temperatures below *T*^unfoldin*g*^, might promote an increase in the enzymatic activity. Nevertheless, NADES addition causes the appearance of enzyme aggregates at high temperatures, as shown in Figure 5B, which have a known effect against activity due to blocking active sites.^63,64^ However, water addition does not influence *T*^unfolding^ of HRP (Figure S2). *T*^unfolding^ values are in the range of what is reported in the literature, namely in the range between 70 and 85 °C.^19,20,22,24,25^ In addition, protein aggregation is observed at temperatures between 80 and 85 °C in the presence of NADES as shown in Figure 5B and Table S7, which was not observed for the control in PBS buffer.

### 4.6 Structural Studies of HRP

**Figure 6:**
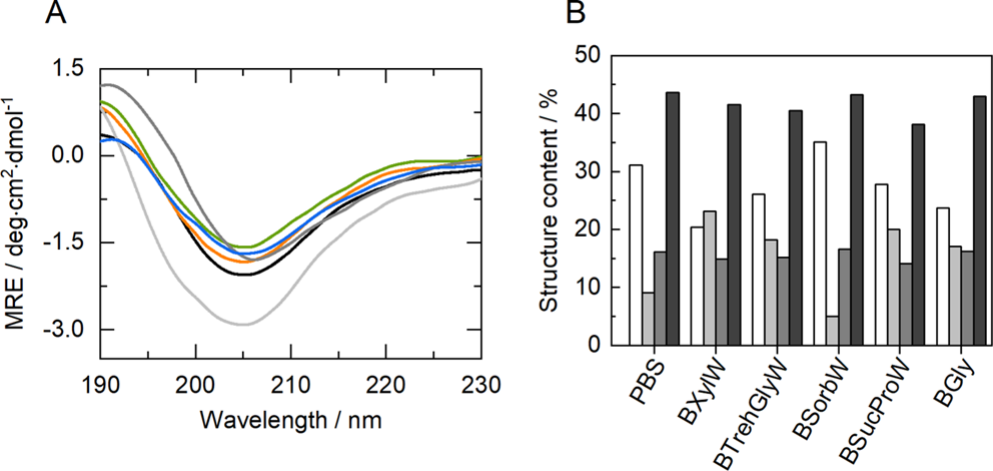
(A) CD spectra of HRP (5 μM) dissolved in PBS (100 mM, pH 7) (black line), as well as BXylW (green line), BTrehGlyW (orange line), BSorbW (light gray line), BSucProW (blue line) and BGly (gray line) solutions; and (B) relative content of secondary structures of HRP in control and NADES: α-helix (white), β-sheet (light gray), turns (dark gray) and random coils (black). Values are reported in Table S8.

The secondary structure of HRP in different solutions was assessed by CD, and measurements in PBS (100 mM, pH 7) were used as a control. Figure 6A compares the HRP’s CD spectra in PBS versus the five NADES-AS herein studied, obtained from 190 to 240 nm. All the CD spectra obtained have similar shapes, with slight intensity differences at 205 nm, which can be attributed to changes in α-helix contents.^65^ It is also possible to observe that there are no signs of protein denaturation, which is usually characterized by a broad negative band below 200 nm.^66^

To obtain more information about the HRP structure, the relative content of each major secondary structure (α-helix, β-sheet and turns) and the random coil of HRP were determined and can be observed in Figure 6B. The CONTIN-LL method was used (via the DICHROWEB web server).^47^ The native HRP structure was the following: 31% α-helix, 9% β-sheet, 16% turns and 44% random coil. It can also be seen in Figure 6B, that the presence of NADES did not significantly change the random coil (∼42%) and turns (∼15%) content of HRP compared to PBS control, according to the information obtained from the spectra analysis. However, it was possible to detect some alterations in the α-helix and β-sheet contents of HRP depending on the NADES in solution. In the presence of BXylW, the contents of α-helix and β-sheet become nearly identical (20% α-helix and 23% β-sheet), while in the presence of BSorbW α-helix was increased at the cost of the decreased β-sheet content (35% α-helix and 5% β-sheet).

HRP is described as a protein with high content of α-helix secondary structure and small β-sheet regions.^2^ Hence, in the presence of BSorbW, the structural changes favored the increase in the α-helix content by 13%, which can be related to the rise in activity (Figure 4). There is evidence that HRP’s activity can be facilitated by higher α-helix and lower β-sheet contents.^43^ As previously demonstrated, a reduction of the relative activity of HRP to 12%, was associated to a decrease in the α-helix structure of c.a. 18%.^65^ More studies show the same relation between loss of activity and drop in the α-helix content of HRP, upon different treatments.^67−69^ These conformational changes are added to positive molecular interactions generated by co-solvents within the protein’s active site, as demonstrated previously in the literature.^9,41^

## Conclusion

In this work, the influence of five betaine-based NADES on the activity and conformation of HRP was studied. First, density, viscosity, and *a*_w_ were measured from 20 to 80 °C at a pressure of 100 kPa. Even though the water mole fractions of the studied systems varied strongly, *a*_w_ values of all the systems were measured to be around 0.4. For the system BGly, the influence of the water content on *a*_w_ was measured. Density and *a*_w_ were modelled with PC-SAFT. PC-SAFT achieves an overall AAD of 0.432 and 7.76% for densities and *a*_w_, respectively. The important conclusion is that binary parameters that were fitted to density were able to predict *a*_w_ values successfully. The ability to use this predictive power of PC-SAFT to characterize *a*_w_ values of NADES will allow the generation of tailor-made solvents for different enzymes in the future, thereby optimizing the design of biocatalytic processes.

As demonstrated in this case study, the presence of NADES in solution, promoted an increase in the thermal and structural stability of HRP. The approach of using NADES in enzyme solutions contributes to a broader insight into biocatalytic reactions in crowded environments and ultimately aims at optimizing the enzymatic environment towards improved stability and efficiency. Overall, an increase in unfolding temperature was observed, and the aggregation appeared at higher temperatures. A transition state before denaturation is promoted by the presence of NADES systems, which could increase enzymatic activity due to the exposure of the heme pocket. On the other hand, the changes in the composition of the secondary structures, α-helix, and β-sheet, show how the protein is restructured in the presence of NADES, by the hydrogen bond network. These conformational changes, more specifically the increase in the α-helix content, increased enzymatic activity with the system BSorbW showing a two-fold increase in HRP’s activity. This improvement reflects the suitability of NADES to be used as efficient co-solvents in biocatalytic reactions as a preserving agent against denaturation and for significant enhanced activity.

## Supporting Information Available

PC-SAFT predictions for all NADES *a*_*w*_; HRP unfolding temperature in BGly + water formulations; PC-SAFT parameters used; PC-SAFT binary interactions parameters used; viscosity values of the NADES under study; viscosity of the BGly + water formulations under study; density of the NADES under study; density of the BGly + water formulations under study; unfolding and aggregation temperatures of HRP; secondary structure composition of HRP (PDF)
