# Supplementary material for: Assessing the Influence of Betaine-Based Natural Deep Eutectic Systems on Horseradish Peroxidase
Source: ACS Sustain Chem Eng. 2022 Sep 12;10(38):12873–81. doi: 10.1021/acssuschemeng.2c04045 (PMC9783073; doi:10.1021/acssuschemeng.2c04045)
Supplement: Supplementary file 1 — sc2c04045_si_001.pdf [file sc2c04045_si_001.pdf]

Supporting Information

Assessing the influence of betaine-based natural deep eutectic systems on Horseradish peroxidase

Nicolás F. Gajardo-Parra †, Liane Meneses ‡, Ana Rita C. Duarte ‡, Alexandre Paiva ‡,, Christoph Held †,***

† Laboratory of Thermodynamics, Department of Biochemical and Chemical Engineering,

TU Dortmund University, Emil-Figge-Str. 70, 44227 Dortmund, Germany.

‡ LAQV-REQUIMTE, Department of Chemistry, School of Science and Technology, NOVA

University Lisbon, 2825-149 Caparica, Portugal.

* abp08838@fct.unl.pt

** christoph.held@tu-dortmund.de

Number of pages – 9

Number of figures – 2

Number of tables – 8

FIGURES

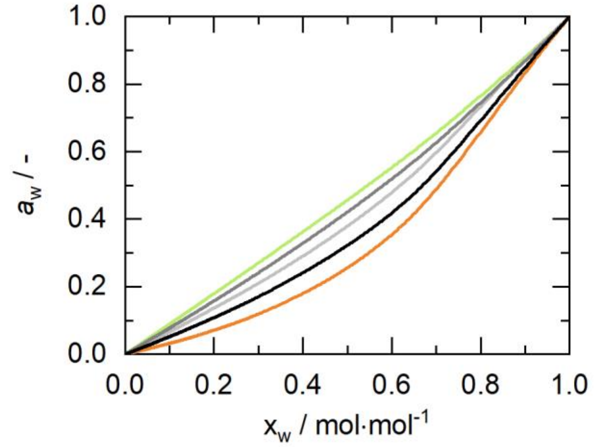

Figure S1: PC-SAFT predictions of a_w for NADES systems, BXylW (orange), BTrehGlyW (light gray), BSorbW (gray), BSucProW (dark gray) and BGly (green).

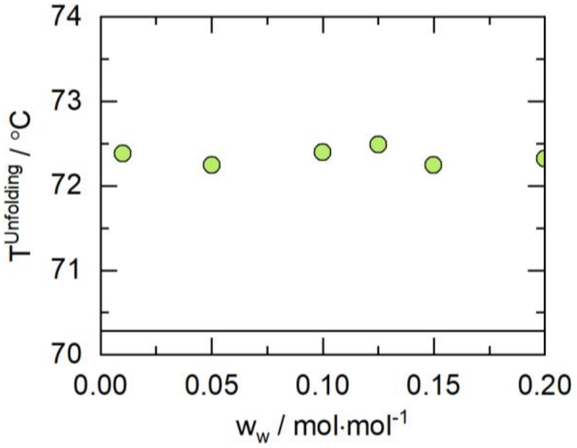

Figure S2: Unfolding temperature (°C) in BGly + water mixtures as function of the water weight fraction: experimental data (green circles). Horizontal continuous black line represents the unfolding temperature in control buffer PBS (100 mM, pH 7).

TABLES

Table S1. PC-SAFT parameters used in this work

Compound	M_w (g/mol)	m_i^{seg}	σ_i (A)	u_i/k_B (K)	N^{sites}	ε^{AiBi}/k_B (K)	κ^{AiBi}	Ref.
Water	18.0153	1.205	*	353.945	1:1	2425.70	0.045099	[1]
Betaine	117.148	8.466	2.547	266.59	1:1	2541.62	0.03840	[2]
Glycerol	92.094	2.007	3.815	430.82	1:1	4633.47	0.00189	[2]
Xylose	150.13	6.012	2.888	283.22	4:4	5000.00	0.10000	[3]
Trehalose	378.33	13.692	2.856	319.85	8:8	5000.00	0.10000	[3]
Sucrose	342.30	14.886	2.827	297.39	8:8	5000.00	0.10000	[3]
Sorbitol	182.17	7.230	2.960	226.94	6:6	5000.00	0.10000	[3]
Proline	115.13	6.7117	2.6562	33.35	1:1	2146.37	0.02350	[4]

* For water, a temperature-dependent segment diameter σ_i=2.7927+10.11· exp(-0.01775·T) - 1.417· exp(0.01146·T) was used.

Table S2. PC-SAFT binary interaction parameters k_{ij} used in this work

Binary System	k_{ij}	Ref.
water-betaine	-0.0922	[2]
water-glycerol	-0.005	[2]
water-xylose	$-0.0342 + 0.000258(T/\mathrm{K} - 298.15\ \mathrm{K})$	[3]
water-sorbitol	$-0.0591 + 0.000093(T/\mathrm{K} - 298.15\ \mathrm{K})$	[3]
water-trehalose	$-0.0274 + 0.000219(T/\mathrm{K} - 298.15\ \mathrm{K})$	[3]
water-sucrose	$-0.0371 + 0.0002560(T/\mathrm{K} - 298.15\ \mathrm{K})$	[3]
water-proline	-0.1235	[4]
betaine-glycerol	$0.219 - 0.0016(T/\mathrm{K} - 298.15\ \mathrm{K})$	This Work*
betaine-xylose	$0.110 - 0.0038\ (T/\mathrm{K} - 298.15\ \mathrm{K})$	This Work*
betaine-sorbitol	$0.200 - 0.001(T/\mathrm{K} - 298.15\ \mathrm{K})$	This Work*
betaine-trehalose	$0.27\text{-}0.001(T/\mathrm{K} - 298.15\ \mathrm{K})$	This Work*
trehalose-glycerol	$0.3\text{-}0.001(T/\mathrm{K} - 298.15\ \mathrm{K})$	This Work*
proline-betaine	$-0.18\text{-}0.01(T/\mathrm{K} - 298.15\ \mathrm{K})$	This Work*

* Fitted to experimental density data between 20°C and 80°C, see section 4.2.

Table S3. Viscosity (mPa·s) of the systems under study at different temperatures and 100 kPa.

	Viscosity (mPa·s)						
	**20 ºC**	**30 ºC**	**40 ºC**	**50 ºC**	**60 ºC**	**70 ºC**	**80 ºC**
**BXylW**	350.05 ± 2.32	174.87 ± 0.79	96.22 ± 0.28	57.10 ± 0.13	36.22 ± 0.06	23.95 ± 0.04	16.81 ± 0.04
**BTrehGlyW**	3392.97 ± 44.04	1354.90 ± 18.41	607.77 ± 7.43	298.54 ± 3.34	161.58 ± 1.59	93.68 ± 0.82	58.15 ± 0.50
**BSorbW**	1523.40 ± 4.43	621.51 ± 4.22	287.84 ± 2.22	147.03 ± 1.15	82.95 ± 0.63	50.18 ± 0.39	32.49 ± 0.25
**BSucProW**	*	673.83 ± 17.68	326.96 ± 8.34	172.21 ± 4.01	99.21 ± 2.09	60.89 ± 1.17	39.80 ± 0.68
**BGly**	2567.53 ± 23.54	1108.33 ± 5.85	529.22 ± 2.30	276.95 ± 0.80	156.61 ± 0.27	94.58 ± 0.05	60.43 ± 0.11

* Solid

Table S4. Viscosity (mPa·s) of the BGly+water mixtures under study at different temperatures and 100 kPa.

	Viscosity (mPa·s)						
x_w	**20 ºC**	**30 ºC**	**40 ºC**	**50 ºC**	**60 ºC**	**70 ºC**	**80 ºC**
**0.087**	2567.53 ± 23.54	1108.33 ± 5.85	529.22 ± 2.30	276.95 ± 0.80	156.61 ± 0.27	94.58 ± 0.05	60.43 ± 0.11
**0.256**	869.97 ± 3.00	407.57 ± 1.98	210.02 ± 1.12	117.73 ± 0.63	70.88 ± 0.37	45.32 ± 0.22	30.51 ± 0.14
**0.421**	275.24 ± 1.45	142.06 ± 0.36	80.01 ± 0.17	48.21 ± 0.08	31.03 ± 0.05	21.05 ± 0.03	14.94 ± 0.03
**0.477**	184.28 ± 0.90	98.55 ± 0.13	57.29 ± 0.03	35.48 ± 0.02	23.39 ± 0.03	16.19 ± 0.02	11.68 ± 0.01
**0.532**	126.55 ± 0.03	69.97 ± 0.02	41.87 ± 0.01	26.58 ± 0.01	17.91 ± 0.01	12.64 ± 0.01	9.29 ± 0.00
**0.598**	64.08 ± 0.12	37.65 ± 0.02	23.77 ± 0.01	15.81 ± 0.00	11.09 ± 0.00	8.11 ± 0.00	6.14 ± 0.00

Table S5. Density (g·cm^{-3}) of the NADES systems under study at different temperatures and 100 kPa.

	Density (g·cm^{-3})						
	**20 ºC**	**30 ºC**	**40 ºC**	**50 ºC**	**60 ºC**	**70 ºC**	**80 ºC**
**BXylW**	1.2211	1.2156	1.2100	1.2044	1.1987	1.1930	1.1871
**BTrehGlyW**	1.2915	1.2851	1.2797	1.2738	1.2679	1.2619	1.2558
**BSorbW**	1.2705	1.2642	1.2585	1.2526	1.2465	1.2405	1.2343
**BSucProW**	*	1.2646	1.2591	1.2535	1.2477	1.2419	1.2361
**BGly**	1.2201	1.2141	1.2084	1.2027	1.1969	1.1912	1.1853

* Solid

Table S6. Density (g·cm^{-3}) of the BGly+water mixtures under study at different temperatures and 100 kPa.

	Density (g·cm^{-3})						
x_w	**20 °C**	**30 °C**	**40 °C**	**50 °C**	**60 °C**	**70 °C**	**80 °C**
**0.087**	1.2201	1.2141	1.2084	1.2027	1.1969	1.1912	1.1853
**0.256**	1.2115	1.2058	1.2000	1.1943	1.1884	1.1826	1.1766
**0.421**	1.1996	1.1939	1.1881	1.1822	1.1763	1.1704	1.1643
**0.477**	1.1945	1.1888	1.1830	1.1771	1.1712	1.1652	1.1591
**0.532**	1.1892	1.1835	1.1777	1.1718	1.1659	1.1599	1.1537
**0.598**	0.7852	0.7815	0.7776	0.7737	0.7697	0.7657	0.7616

Table S7. Unfolding temperatures of HRP in the presence of NADES systems compared to control buffer and aggregation temperatures of HRP.

Binary System	$T^{on\text{-}set}$ / °C	Δ $T^{unfolding}$ / °C	$T^{Aggregation}$ / °C
PBS	57.1	-	-
BXylW	54.5	3.59	81.0
BTrehGlyW	55.9	3.67	85.6
BSorbW	39.8	5.18	81.4
BSucProW	40.6	2.89	82.1
BGly	52.1	2.50	85.7

Table S8. Secondary structure composition (%) of HRP upon NADES systems addition.

System	α-helix	β-sheets	Turns	Random coils
PBS	31.1	9.1	16.1	43.6
BXylW	20.4	23.1	14.9	41.5
BTrehGlyW	26.1	18.2	15.2	40.5
BSorbW	35.1	5.0	16.6	43.2
BSucProW	27.8	20.0	14.1	38.1
BGly	23.7	17.1	16.2	43.0

REFERENCES

[1] L.F. Cameretti, G. Sadowski, Modeling of aqueous amino acid and polypeptide solutions with PC-SAFT, Chemical Engineering and Processing: Process Intensification 47 (2008) 1018–1025. https://doi.org/10.1016/j.cep.2007.02.034.

[2] C. Held, G. Sadowski, Compatible solutes: Thermodynamic properties relevant for effective protection against osmotic stress, Fluid Phase Equilibria 407 (2016) 224–235. https://doi.org/10.1016/j.fluid.2015.07.004.

[3] C. Held, G. Sadowski, A. Carneiro, O. Rodríguez, E.A. Macedo, Modeling thermodynamic properties of aqueous single-solute and multi-solute sugar solutions with PC-SAFT, AIChE J. 59 (2013) 4794–4805. https://doi.org/10.1002/aic.14212.

[4] B.-S. Lee, K.-C. Kim, Study on the activity coefficients and solubilities of amino acids in aqueous solutions with perturbed-chain statistical associating fluid theory, Korean J. Chem. Eng. 27 (2010) 267–277. https://doi.org/10.1007/s11814-009-0351-z.
